# Contributions of *cis-* and *trans-*Regulatory Evolution to Transcriptomic Divergence across Populations in the *Drosophila mojavensis* Larval Brain

**DOI:** 10.1093/gbe/evaa145

**Published:** 2020-07-11

**Authors:** Kyle M Benowitz, Joshua M Coleman, Carson W Allan, Luciano M Matzkin

**Affiliations:** e1 Department of Entomology, University of Arizona; e2 Department of Biological Sciences, University of Alabama in Huntsville; e3 Department of Ecology and Evolutionary Biology, University of Arizona; e4 BIO5 Institute, University of Arizona

**Keywords:** cactophilic, local adaptation, pleiotropy, RNA-seq, transcriptional regulation

## Abstract

Natural selection on gene expression was originally predicted to result primarily in *cis*- rather than *trans*-regulatory evolution, due to the expectation of reduced pleiotropy. Despite this, numerous studies have ascribed recent evolutionary divergence in gene expression predominantly to *trans*-regulation. Performing RNA-seq on single isofemale lines from genetically distinct populations of the cactophilic fly *Drosophila mojavensis* and their F_1_ hybrids, we recapitulated this pattern in both larval brains and whole bodies. However, we demonstrate that improving the measurement of brain expression divergence between populations by using seven additional genotypes considerably reduces the estimate of *trans*-regulatory contributions to expression evolution. We argue that the finding of *trans*-regulatory predominance can result from biases due to environmental variation in expression or other sources of noise, and that *cis*-regulation is likely a greater contributor to transcriptional evolution across *D. mojavensis* populations. Lastly, we merge these lines of data to identify several previously hypothesized and intriguing novel candidate genes, and suggest that the integration of regulatory and population-level transcriptomic data can provide useful filters for the identification of potentially adaptive genes.

**Significance**

Gene expression evolution can be driven by changes in the focal gene itself (*cis*-regulation) or by changes in the genes that regulate it (*trans*-regulation). The importance of these two processes, and their contribution to adaptation specifically, remains under debate. Through a novel integration of data on genetic variation in gene expression with data on gene regulatory evolution, we found increased evidence for a primary role of *cis*-regulation in both total expression evolution as well as adaptive expression evolution. These results inject nuance into the discussion of how regulatory processes influence evolution within species and outline an approach for using expression data to address adaptive hypotheses.

## Introduction

Statistical correlations between phenotypes impose fundamental constraints on phenotypic evolution (Lande 1979). As such, selection may disfavor the propagation of especially pleiotropic mutations whose causal effects alter many traits (Otto 2004). This idea has led to considerable speculation on the precise molecular effects of successful mutations. Vigorous debate regarding the relative importance of coding sequence and gene regulatory evolution hinged on claims regarding the respective pleiotropic consequences of these types (Hoekstra and Coyne 2007; Carroll 2008). Within the category of regulatory mutations, however, further distinctions are likely to be relevant in this context. Specifically, *trans*-regulatory changes, which are primarily a consequence of changes in expression and/or structure of transcription factors, are expected to affect large networks of target genes and therefore be highly pleiotropic (Gibson 1996; Wittkopp 2007; but see Lynch and Wagner [2008]). In contrast, *cis*-regulatory mutations, occurring in promoters or enhancers of the target genes themselves, might affect only single genes in specific contexts (Stern 2000; Prud’homme et al. 2007). In an early and thorough theoretical treatment of the subject, Wray et al. (2003) did not equivocate in hypothesizing that *cis*-regulatory evolution should primarily be responsible for the evolution of gene expression phenotypes.

In the years since that prediction, the accumulation of evidence regarding the prevalence of *cis*- and *trans*-regulatory effects in evolution has led to a far murkier picture. This may in part reflect the methodological diversity of studies approaching the question (reviewed in Signor and Nuzhdin [2018]). Some experiments, such as chromosomal substitutions (Hughes et al. 2006; Osada et al. 2006), crosses utilizing the diversity of a reference panel (Genissel et al. 2007; Fear et al. 2016; Osada et al. 2017), and eQTL mapping studies (Massouras et al. 2012; King et al. 2014) have generally, but not always (Lemos et al. 2008; Wang et al. 2008) corroborated the hypothesis, finding greater contributions of *cis*-effects to intrapopulation variation. On the other hand, results from another frequently used experimental design, which we will henceforth call the F_1_ hybrid design, have consistently led to the opposite conclusion. The F_1_ hybrid design requires expression data from two parental lines and their F_1_ hybrids. *Cis*-regulatory effects are measured using the differential expression of allele-specific reads within the hybrid samples, whereas *trans*-regulatory effects are calculated by subtracting the *cis*-regulatory effect from the overall differential expression between the parental lines (Wittkopp et al. 2004). Usage of the F_1_ hybrid design has repeatedly found that *trans*-regulation dominates expression variability within species, whereas *cis*-regulation plays a greater role in interspecific differences (Graze et al. 2009; Wittkopp et al. 2004, 2008; McManus et al. 2010; Suvorov et al. 2013; Coolon et al. 2014; Metzger et al. 2017; Glaser-Schmitt et al. 2018).

Given the power of the F_1_ hybrid design and its applicability to a wide range of study systems and biological contexts, closer attention to the interpretations stemming from this approach is merited. As such, recent work has begun to approach the F_1_ hybrid paradigm with increased nuance. Glaser-Schmitt et al. (2018) perform a tissue-specific study, filling an important gap given the focus of previous work on whole-body samples. Taking this one step further, Combs and Fraser (2018) estimate fine-scale spatial variation in allele-specific expression within embryos. From a different angle, two recent commentaries (Fraser 2019; Zhang and Emerson 2019) make salient points regarding potential biases in the estimation of *trans*-regulatory divergence given that it cannot be estimated independently of *cis*-regulatory and parental divergence using this approach, and stress the need for replication to mitigate this. Here, we build from these efforts and probe the initial findings from an across-population F_1_ hybrid study using two simple experiments. First, we conduct a tissue-specific study in parallel with a whole-body study, to directly estimate the effects of sample heterogeneity on the estimation of regulatory type. Second, we supplement our measures of parental divergence with further sampling of genotypes from each parental population, to gain more confidence in patterns of within and between-population variation in transcription.

We apply these experiments to an investigation of gene expression evolution in larval brains across two populations of the cactophilic fly *Drosophila mojavensis*. This combination of organism and tissue lends itself to a strong hypothesis of predominant *cis*-regulatory evolution, for two reasons. First, *D. mojavensis* is predicted to have experienced strong differential selection pressures across populations due to variable ecological conditions. The two populations studied here, from Santa Catalina Island, CA, and the Sonoran Desert (Guaymas, Sonora, Mexico and Organ Pipe National Monument, Arizona), are genetically distinct (Reed et al. 2006) primarily utilize highly divergent cactus species, the prickly pear *Opuntia littoralis* and the columnar *Stenocereus thurberi*, respectively (Heed 1978; Ruiz et al. 1990). These host cacti form unique chemical and nutritional environments (Kircher 1982; Starmer and Phaff 1983), and detoxification genes in particular have seen substantial expression and coding sequence evolution across these populations (Matzkin et al. 2006; Allan and Matzkin 2019). In addition to selection from the host, these populations experience vastly different temperature and humidity regimes, which is expected to generate selection broadly on phenology and organismal physiology (Matzkin 2014). We choose to focus on brains here in part because we previously identified larval behavioral differences related to locomotion and pupation (Coleman et al. 2018), indicating the potential for the evolution of expression changes in the brain, as well as muscle and fat body. Second, despite this potential for selection, there are also a priori expectations that transcriptome-wide evolution should actually be reduced. Brain gene expression is highly conserved in many animals, including *Drosophila* (Brawand et al. 2011; Catalán et al. 2012; Uebbing et al. 2016). Additionally, gene expression in larvae is more conserved than in later developmental stages in *Drosophila* (Artieri and Singh 2010). The pairing of strong directional and strong stabilizing selection across genes is precisely the scenario that should result in transcriptional fine-tuning due to *cis*-regulatory evolution. Thus, our expectation was to uncover a greater role for *cis*-regulatory changes than observed in other intraspecific studies using similar experimental designs.

## Materials and Methods

### Sample Collection and Sequencing

For initial analysis of population divergence and analyses of allele-specific expression, we used single genome-sequenced isofemale lines of *D. mojavensis* from Santa Catalina Island, CA (Drosophila 12 Genomes Consortium 2007) and Guaymas, Sonora, Mexico (Allan and Matzkin 2019). These lines have been maintained as isofemale lines without direct inbreeding in the laboratory on banana-molasses media (Coleman et al. 2018) since 2002 and 1999, respectively. We generated F_1_ hybrids between these two lines by placing 20 virgin genome-line Catalina Island males and 20 virgin genome-line Sonora females in vials containing banana-molasses media, and performed the reciprocal cross in an identical manner. For analyses of genotypic variation in expression, we selected seven additional isofemale lines from Santa Catalina Island and seven isofemale lines from the Sonoran Desert population from Organ Pipe National Monument, AZ, which were collected between 2007 and 2009 (Coleman et al. 2018) and maintained as described earlier.

We collected all samples during the third-instar wandering stage. For whole-body samples, we collected five larvae per replicate, washing each larva in deionized water before storing them on ice in tris–EDTA buffer. For brain samples, we dissected ten brains per replicate in tris–EDTA before storing them on ice. We then froze samples at −80 °C for storage. We collected three biological replicates for each genome line and hybrid (brain and body) and single replicates of each additional isofemale line (brain only). We ground samples in TRIzol (Thermo Fisher, Waltham, MA) and used Qiagen RNEasy columns (Qiagen, Hilden, Germany) to extract RNA, prepared libraries using Illumina TruSeq kits (Illumina, San Diego, CA), and sequenced samples as 150-bp paired-end reads on an Illumina HiSeq. Information on sample identity and sequencing can be found in supplementary table S1, Supplementary Material online.

### Bioinformatic Analysis

We removed Illumina adapters and low-quality sequence using Trimmomatic (Bolger et al. 2014) and used NextGenMap (Sedlazeck et al. 2013) with default parameters to separately map all reads to both the original Catalina Island genome (Drosophila 12 Genomes Consortium 2007; FlyBase version r1.04_FB2018_06) and the same genome templated with Sonora genomic reads (Allan and Matzkin 2019). We calculated total read counts at the gene level for each sample using HTSeq-count (Anders et al. 2015), using the reads mapped to the Catalina Island genome for analysis. We then downsampled reads to 11,908,854 reads over 13,410 genes in brain samples and 14,001,634 reads over 13,628 genes in whole-body samples, to match the lowest coverage sample in each tissue. For the additional brain isofemale lines, which were more highly covered, we downsampled to 18,665,415 reads over 13,565 genes. From these gene sets, we analyzed only genes with at least ten total reads in each sample. After comparing the consistency between biological replicates within each group using Spearman’s correlation coefficients, we discarded three samples from the genome lines as outliers: one Sonora brain sample, one Sonora (f)×Catalina Island (m) hybrid body sample, and one Catalina Island (f)×Sonora (m) body sample. In the analysis of genotypic variation in the brain, we discarded an additional Sonora sample as an outlier based on the same criteria. We included only a single randomly chosen replicate from each genome-sequenced line in the analysis of genotypic variation to avoid pseudoreplication.

For allele-specific counts, we used SAMtools mpileup (Li et al. 2009) and VarScan2 (Koboldt et al. 2013) to identify informative variants for allele-specific expression analysis. We first removed all SNPs where we found, in any of the parental genotype samples the allele from the other parental genome at >5% frequency (Glaser-Schmitt et al. 2018). This step helps to avoid analyzing heterozygous sites, which will lead to inaccuracy in the assignment of reads to parental genomes. We then compared the remaining SNPs from the mapping results to both reference genomes and removed sites with substantially differing allele frequencies in the two resulting data sets, following previous work (Benowitz et al. 2019). In this way, we removed sites potentially affected by mapping bias, which, although not a major problem here (supplementary table S1, Supplementary Material online), would result in overestimation of allele-specific expression of genes containing those sites. We then filtered all bam files (mapped to the Catalina Island reference) for informative reads using VariantBam (Wala et al. 2016) and output these reads as text files using sam2tsv (https://lindenb.github.io/jvarkit/Sam2Tsv.html; last accessed July 2020). We then counted allele-specific reads overlapping each informative SNP, generating gene-level counts after accounting for reads overlapping multiple variants in R 3.4 (R Core Team 2018). We ran this pipeline independently for brain and body samples. We randomly downsampled allele-specific reads in each brain sample to 4,541,638 reads, matching the reads in the lowest coverage sample. These reads covered SNPs in 7,933 genes. For the whole-body samples, which covered 8,584 genes, we downsampled all read counts to 4,939,804 total reads, to preserve the ratio of reads per gene between brains and whole bodies. In both data sets, we analyzed only genes containing at least ten total reads in each sample. The same three samples identified as outliers above were also outliers in this data set and we accordingly discarded them for allele-specific expression analysis as well.

### Statistical Analysis

We calculated per-gene parental divergence from the total (not allele-specific) expression counts as log_2_(*P*_CI_/*P*_SON_), where *P*_CI_ and *P*_SON_ are either parental genome-sequenced line means (when comparing single genotypes; in brains and whole bodies) or parental population means (when comparing all genotypes; brains only). We calculated transcriptome-wide differentiation across populations as 1−*ρ*, where *ρ* is the Spearman’s correlation of expression divergences between populations. We estimated 95% confidence intervals of 1−*ρ* from 10,000 bootstrapped replicates.

We calculated *cis*-regulatory divergence, following previous studies, as log_2_(*H*_CI_/*H*_SON_), where *H*_CI_ and *H*_SON_ are averages of allele-specific counts across all F_1_ hybrid replicates. We then calculated *trans*-regulatory divergence as the difference between parental divergence (between the genome-sequenced lines) and *cis*-regulatory divergence, log_2_(*P*_CI_/*P*_SON_)−log_2_(*H*_CI_/*H*_SON_). We independently assessed the contributions of *cis*- and *trans*-regulatory divergence to both metrics of parental divergence using Spearman’s correlation coefficient *ρ*. As above, we assessed 95% confidence intervals from 10,000 bootstrap replicates. We visualized correlations using least-squares regression lines and 95% confidence regions around those regressions using the R package ggplot2.

We estimated differential expression between parental populations using FDR-corrected negative binomial tests using the R package NBPseq (Di et al. 2011). We also used negative binomial tests comparing allele-specific counts to assess the significance of *cis*-regulatory effects. To estimate the significance of *trans*-regulatory effects at the gene level, we used Fisher’s exact tests comparing the ratio of allele-specific expression differences to total gene expression differences in the parental samples.

For evolutionary analysis of regulatory evolution in the brain, we used previously published d*N*/d*S* values across the four *D. mojavensis* populations (Allan and Matzkin 2019). To estimate network connectivity, we identified the closest *Drosophila melanogaster* ortholog for each gene and used the in-degree metric calculated in Marbach et al. (2012) and used previously in a similar analysis in Yang and Wittkopp (2017). Briefly, in-degree quantifies the number of transcription factors found to have significant regulatory interactions with each gene. We analyzed d*N*/d*S* and in-degree between genes in different regulatory categories using Mann–Whitney *U* test with the R function pairwise.wilcox.test, using Holm’s method to correct for multiple comparisons. For these analyses, we defined *trans-*regulated genes via the brain analysis using multiple parental genotypes. We performed all statistical analyses in R 3.4 (R Core Team 2018).

## Results

### Analysis of Single Parental Genotypes in Brains and Whole Bodies

Examining a single genotype per population, transcriptome-wide expression differentiation across populations was lower in brains (1−*ρ*  =  0.017; 95% CI = [0.016, 0.018]) than in bodies (1−*ρ*  =  0.036; 95% CI = [0.034, 0.038]), as expected. In contrast, we found evidence for considerably more significantly differentially expressed (DE) genes in brains than in bodies (table 1 and supplementary table S2, Supplementary Material online). The lack of statistical support for many DE genes in bodies despite increased overall expression differences reflects substantially greater intragenotypic variation in whole-body data (supplementary fig. S1, Supplementary Material online).

**Table 1:** Numbers of Differentially Expressed, *Cis*-Regulated, and *Trans*-Regulated Genes Using Both the Single-Genotype Parental Data Set and the Multigenotype Parental Data Set (brains only)

	Single Parental Genotype		Multiple Parental Genotypes
	Whole Bodies	Brains	Brains
DE genes	231	530	308
*Cis*-regulated genes	20	143	–
*Trans*-regulated genes	1072	467	265

Note.—The calculation of *cis*-regulated genes relies only on F_1_ hybrids, thus there is no recalculation of the number of *cis*-regulated genes in the multiparent genotype data set.

**Fig. 1.**
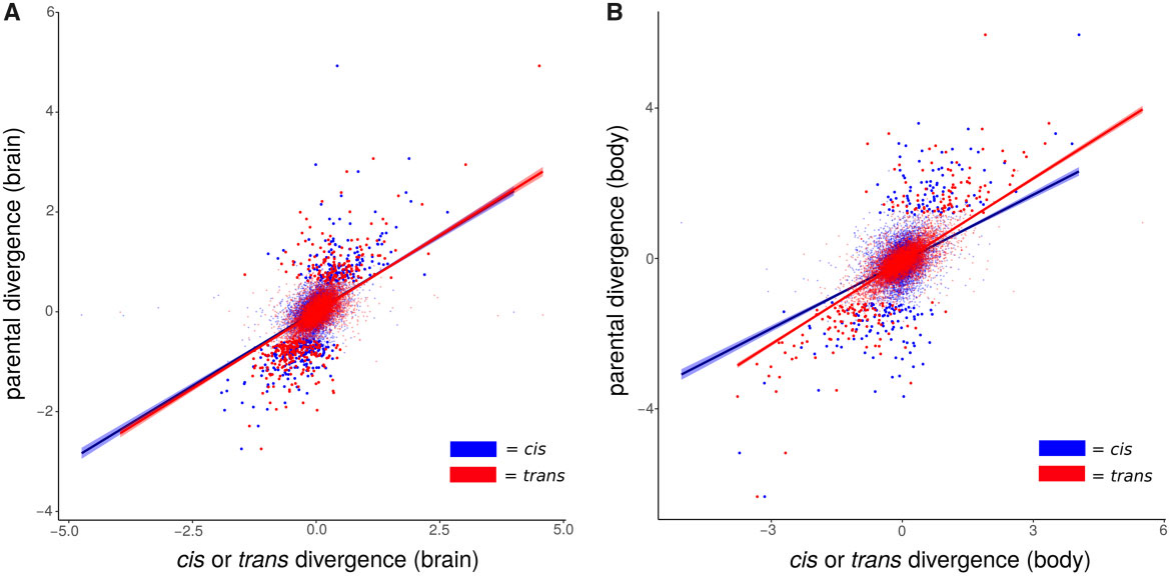
The relationship between *cis*- and *trans*-regulatory divergence and divergence between parental genotypes in (*A*) brains and (*B*) whole bodies. Bold points indicate significantly differentially expressed genes in each data set. Trend lines represent least-squares regressions surrounded by 95% confidence intervals.

We then correlated measures of divergence across all genes with measures of *cis*- and *trans*-regulatory divergence as estimated from F_1_ hybrids between these two lines (Coolon et al. 2014; Metzger et al. 2017). This correlation broadly estimates the contributions of each regulatory type to total expression divergence without relying on thresholds of statistical significance. We found that *trans*-effects were more closely associated with parental divergence than *cis*-effects to parental divergence in both brains (*trans*: *ρ*  =  0.577; 95% CI = [0.561, 0.591], *cis*: *ρ*  =  0.455; 95% CI = [0.438, 0.472], fig. 1*A*) and whole bodies (*trans*: *ρ*  =  0.643; 95% CI = [0.629, 0.657], *cis*: *ρ*  =  0.338; 95% CI = [0.319, 0.356], fig. 1*B*). Lastly, we found more individual genes displaying evidence of regulation in *trans* than in *cis* in both brains and bodies, although this trend was much more dramatic in whole bodies (table 1 and supplementary tables S3 and S4, Supplementary Material online).

### Analysis of Multiple Parental Genotypes in Brains

**Fig. 2.**
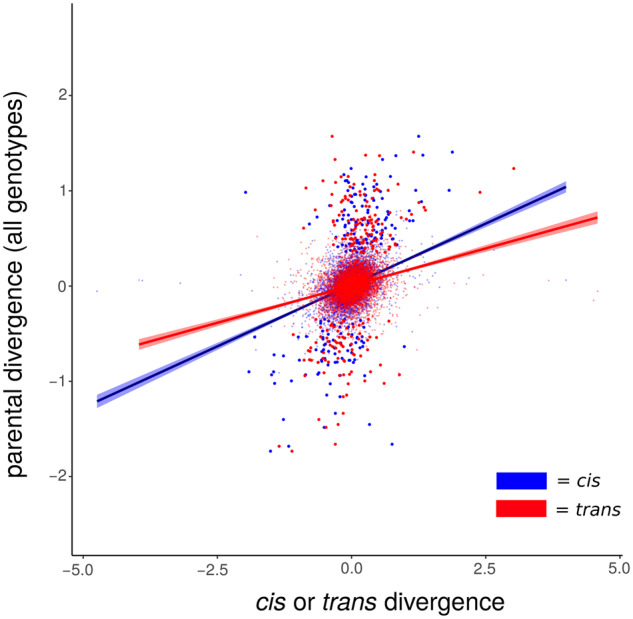
The relationship between *cis*- and *trans*-regulatory divergence and divergence between parental genotypes in brains, where parental divergence is measured using all genotypes. *Cis*- and *trans*-regulatory divergence data are the same as in figure 1. Bold points indicate significantly differentially expressed genes. Trend lines display least-squares regressions surrounded by 95% confidence intervals.

The above comparison, using only single parental genotypes, provides a limited estimate of expression evolution across populations. To more confidently assess expression evolution across populations, we analyzed brain RNA-seq data from seven additional Catalina Island genotypes and six additional Sonoran genotypes. Specifically, we expected the inclusion of multiple genotypes to reduce sampling error and result in lower expression differentiation between populations. Indeed, parental divergence across populations was lower in this data set (1−*ρ*  =  0.005; 95% CI = [0.004, 0.005]) and the number of significantly DE genes was reduced (table 1 and supplementary table S2, Supplementary Material online). We then examined correlations between parental divergence using multiple genotypes with the identical *cis*- and *trans*-regulatory divergence values calculated above. We now found the opposite result: *cis*-effects were more related to population divergence as measured by multiple genotypes than *trans*-effects (*cis*: *ρ*  =  0.362; 95% CI = [0.343, 0.380], *trans*: *ρ*  =  0.287; 95% CI = [0.268, 0.306], fig. 2). We also used the multigenotype data set to recalculate the number of *trans-*regulated genes, and found far fewer than in the single genotype analysis, although still considerably more than the number of *cis-*regulated genes (table 1 and supplementary tables S3 and S4, Supplementary Material online).

### Evolutionary and Candidate Gene Analysis

**Fig. 3.**
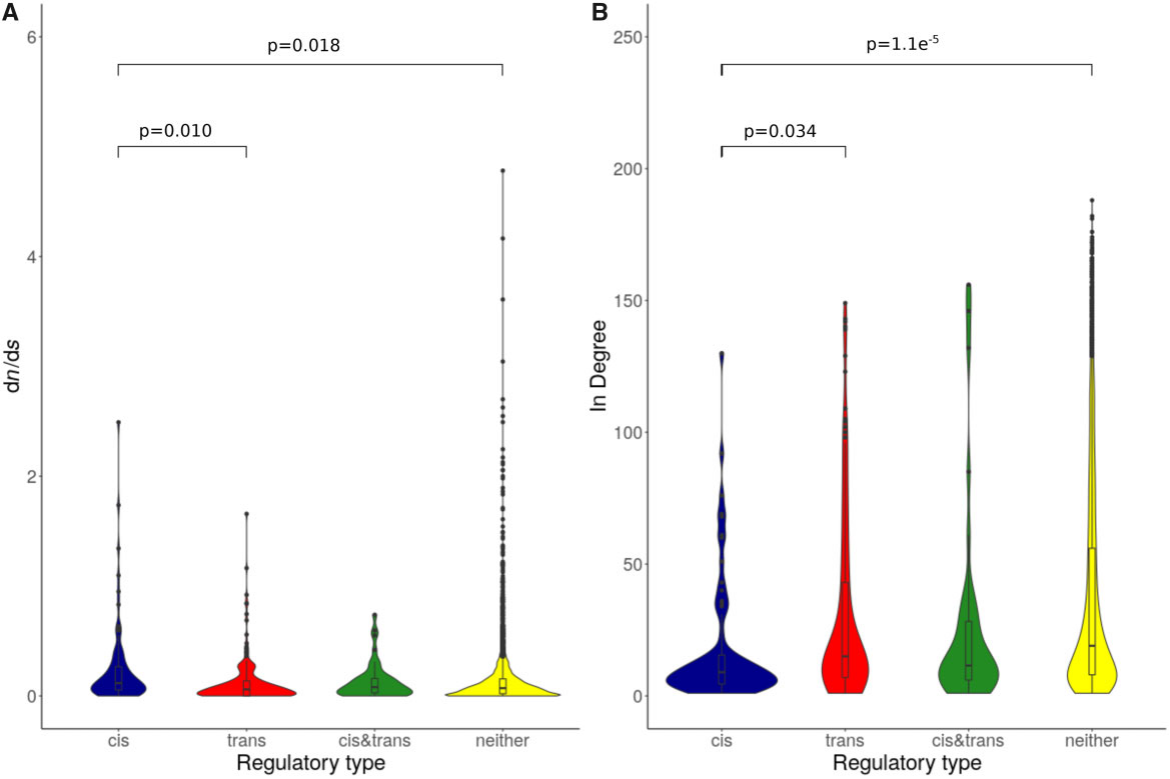
Relationships between regulatory classification and gene-level metrics. (*A*) Comparison of evolutionary rate (d*N*/d*S*) among *Drosophila mojavensis* populations (from Allan and Matzkin [2019]). (*B*) Comparison of the number of transcriptional regulators (in degree) as inferred from *D. melanogaster* orthologs (Marbach et al. 2012). *P* values for significant pairwise comparisons are indicated, all other comparisons are nonsignificant.

We found that *cis*-regulated genes had higher evolutionary rates within *D. mojavensis* than did *trans*-regulated genes (*P* = 0.0010) or genes that were either conserved or lacking a clear regulatory pattern (*P* = 0.0018). We also found that the in-degree (number of transcriptional regulators), as inferred from *D. melanogaster* orthologs, of *cis*-regulated genes in our data set was lower than that of either *trans*-regulated genes (*P* = 0.0034) or those with no identified regulatory type (*P* = 1.1e^−5^)*.* The distributions of d*N*/d*S* and in-degree values for genes in each regulatory classification are shown in figure 3. To examine potential evolutionary hypotheses on a more granular level, we also compiled a list of candidate genes displaying two criteria: differential expression between the two populations in the multiple genotype brain data set, and a statistically significant pattern of *cis-* and/or *trans*-regulatory evolution. About 68 genes met these criteria, of which 27 where *cis*-regulated, 35 were *trans*-regulated, and six had significant *cis-* and *trans* effects (table 2). Of these six, five showed evidence for compensatory (*cis*×*trans*) evolution, whereas only one showed evidence for combined (*cis*+*trans*) evolution.

**Table 2:** The Genes Displaying Both Differential Expression across Populations in the Brain Multigenotype Data Set and Statistical Evidence for *Cis-* and/or *Trans*-Regulatory Evolution in the Brain

*Drosophila mojavensis* FlyBase ID	*Drosophila melanogaster* Gene Name	*P* Value (Parental DE)	*P* Value (Regulatory Type)	Population with Higher Expression	Regulatory Classification
FBgn0140302	Cyp28a5	6.75E-30	4.3E-10	SON	*cis*
FBgn0147102	GstD1	1.67E-28	8.25E-09	CI	*cis*
FBgn0139804	Ugt36Bc	2.81E-22	5.14E-08	SON	*cis*
FBgn0280601	NA	2.62E-19	0.00000471	CI	*cis*
FBgn0143628	NA	1.22E-16	0.00159	SON	*cis*
FBgn0147447	Cyp9f2	7.89E-13	0.000000667	CI	*cis*
FBgn0136497	CG14567	2.41E-11	5.24E-10	CI	*cis*
FBgn0133237	RdhB	1.82E-08	2.58E-08	CI	*cis*
FBgn0133140	CG5379	6.73E-08	0.000332	SON	*cis*
FBgn0136870	CG33521	0.000000535	0.00000103	SON	*cis*
FBgn0136131	CG2211	0.0000014	0.000000357	CI	*cis*
FBgn0146788	CG18547	0.0000025	0.0195	SON	*cis*
FBgn0140494	CG31777	0.0000444	0.0000392	SON	*cis*
FBgn0139666	CG5316	0.0000474	1.67E-09	SON	*cis*
FBgn0136235	CG33969	0.0000835	0.000765	CI	*cis*
FBgn0142519	Phr	0.000162	0.00133	SON	*cis*
FBgn0145297	CG10550	0.000917	0.00377	SON	*cis*
FBgn0140061	NA	0.00149	2.15E-11	SON	*cis*
FBgn0147596	CG34409	0.00373	0.0169	CI	*cis*
FBgn0146770	Cyp12e1	0.00447	0.000129	CI	*cis*
FBgn0085888	ST6Gal	0.00738	0.009	SON	*cis*
FBgn0142451	CG9344	0.00885	0.0111	CI	*cis*
FBgn0280848	NA	0.0199	0.0486	SON	*cis*
FBgn0136999	CG8086	0.0208	0.00551	SON	*cis*
FBgn0132957	CG10165	0.021	0.00602	CI	*cis*
FBgn0141924	CG3511	0.0244	0.0322	CI	*cis*
FBgn0132961	Acyp2	0.0434	0.0195	CI	*cis*
FBgn0134299	Fbp1	3.37E-26	1.72E-46	CI	*trans*
FBgn0145801	Lsp1beta	4.18E-24	6.82E-35	CI	*trans*
FBgn0143382	Lsp2	7.92E-18	2.74E-22	CI	*trans*
FBgn0142713	Sans	1.53E-16	0.00527	SON	*trans*
FBgn0140799	Lsp1gamma	1.71E-16	2.23E-08	CI	*trans*
FBgn0140406	Fbp2	1.35E-12	2.53E-15	CI	*trans*
FBgn0135991	NA	2.29E-12	0.0000427	CI	*trans*
FBgn0135183	CAH2	4.69E-12	0.000012	CI	*trans*
FBgn0134843	CG32037	2.93E-10	0.0223	CI	*trans*
FBgn0139371	GstE2	1.4E-09	0.00239	CI	*trans*
FBgn0146682	TyrRII	2.96E-09	0.00385	SON	*trans*
FBgn0139800	Mhc	3.12E-09	0.0346	SON	*trans*
FBgn0139723	Cg25C	8.58E-09	0.00847	CI	*trans*
FBgn0138516	Sirup	2.74E-08	0.0179	SON	*trans*
FBgn0147600	MtnA	0.000000896	4.35E-39	CI	*trans*
FBgn0142365	CG7997	0.00000124	0.000746	CI	*trans*
FBgn0141762	CG3520	0.0000019	0.000176	CI	*trans*
FBgn0142390	Bru	0.0000187	0.000000015	SON	*trans*
FBgn0147345	CG14291	0.0000735	0.0226	SON	*trans*
FBgn0141634	CG30460	0.000217	0.00153	SON	*trans*
FBgn0146346	NA	0.000343	0.00903	CI	*trans*
FBgn0142161	CG13742	0.000549	0.000399	CI	*trans*
FBgn0146625	CG31278	0.00111	0.0197	CI	*trans*
FBgn0138139	Nocte	0.00196	2.85E-33	CI	*trans*
FBgn0132831	CG6364	0.00233	0.00353	CI	*trans*
FBgn0134066	CG18081	0.00795	0.000801	CI	*trans*
FBgn0146517	Snap25	0.0111	0.0000444	CI	*trans*
FBgn0140557	CG17124	0.0118	0.00327	CI	*trans*
FBgn0138450	CG14785	0.0178	0.00243	SON	*trans*
FBgn0132826	CG6723	0.0179	0.0000984	SON	*trans*
FBgn0143281	RpL23	0.0267	0.00682	CI	*trans*
Fbgn0145013	CG34377	0.027	0.000183	CI	*trans*
FBgn0144093	NA	0.0313	0.00176	SON	*trans*
FBgn0145765	Npc2b	0.0363	0.000000643	SON	*trans*
FBgn0145063	Obp99b	0.0397	0.0289	CI	*trans*
FBgn0146132	Obp99a	1.67E-28	*cis*: 4.74E-04	CI	*cis + trans*
			*trans*: 4.30E-06		
FBgn0141410	CG18067	2.95E-11	*cis*: 7.01E-13	SON	*cis x trans*
			*trans*: 1.12E-04		
FBgn0146495	Spartin	0.0000296	*cis*: 7.89E-03	CI	*cis x trans*
			*trans*: 6.74E-11		
FBgn0084818	NA	0.00145	*cis*: 1.02E-05	CI	*cis x trans*
			*trans*: 7.85E-04		
FBgn0147549	NANS	0.0039	*cis*: 2.13E-03	SON	*cis x trans*
			*trans*: 1.03E-05		
FBgn0141293	CG15651	0.0235	*cis*: 4.28E-16	SON	*cis x trans*
			*trans*: 1.11E-06		

## Discussion

The measurement of allele-specific expression in F_1_ hybrid offspring has been one of the primary approaches for understanding genome-wide patterns of *cis*- and *trans*-regulatory evolution both within and between species. Although other methodologies for quantifying these effects have been used effectively, the advantage of the F_1_ hybrid approach, in our opinion, is its simplicity and potential applicability to a wide range of study systems and evolutionary contexts. However, as with any other genome-scale approach to evolution, the conclusions stemming from F_1_ hybrid studies come with biases and limitations. Here, our goal was to investigate how two straightforward modifications to this common experimental design affect the evolutionary interpretations regarding the prevalence of *cis*- and *trans*-regulation in natural populations. Furthermore, we aimed to leverage the ecological and evolutionary information from our model system, *D. mojavensis*, to examine how successfully the integration of complex regulatory data can uncover adaptive gene expression changes across populations.

### The Effects of Tissue Specificity on Estimation of Regulatory Type

Most of the original genome-wide studies of *cis*- and *trans*-regulatory evolution used gene expression measurements taken from whole organisms. This experimental design may blunt the ability to detect *cis*-regulatory changes, if those changes are only realized in a subset of tissues. Here, we performed allele-specific expression experiments in both whole bodies and brains of larval *D. mojavensis* in parallel, to determine if and how much the use of heterogeneous tissue samples affects quantification of regulatory type. We found clear evidence that our analysis of whole-body samples both overestimated *trans* effects and underestimated *cis* effects. This is reflected both in correlations between regulatory and parental divergences (fig. 1) as well as in the numbers of genes statistically categorized as *cis-* or *trans-*regulated (table 1). We cannot say precisely how much of this difference is due to the problems of using heterogeneous tissue samples and how much is due to regulatory properties specific to the larval brain. A systematic data set of allele-specific expression in multiple tissues and life stages collected would be needed to robustly address this question.

### The Effects of Using Multiple Parental Genotypes on Estimation of Regulatory Type

It is well known that the F_1_ hybrid approach can bias estimates of *trans*-regulatory evolution, because they cannot be estimated independently of the measurement of parental expression divergence. Fraser (2019) pointed out how this issue, when combined with error in the estimation of allele-specific expression, can lead to overestimation of *cis–trans* compensatory evolution. By the same logic, we hypothesized that simple errors in the measurement of parental expression evolution might lead to inflation of the degree of *trans*-regulatory effects.

To address this issue, we simply compared the quantification of *cis-* and *trans-*regulatory effects in brains using two measures of parental expression divergence across populations: one measured from only the single genotype utilized in the allele-specific expression experiment, and one using seven additional genotypes from each population. Using population expression values has two obvious potential consequences for each gene. First, it should reduce noise in the estimation of population expression means coming from the small sample size of using only a few samples of a single genotype. This should lead to reduced estimates of *trans*-regulatory evolution. However, there will also be a subset of genes whose expression in the focal genotype substantially differs from the mean of its population. Thus, for some number of genes, our method should result in the artificial detection of *trans*-regulatory effects because the parental divergence will be mismatched with the allele-specific expression data.

Despite this, we find that using estimates of parental population divergence using multiple genotypes considerably reduces the genome-wide estimate of *trans*-regulatory evolution. Our data present mixed results, however, on the question of whether *cis*- or *trans*-regulation is primarily responsible for expression evolution between our populations. Although the correlation analysis suggests that *cis*-regulatory divergence is more closely related to population divergence (fig. 2), our per-gene hypothesis tests maintain nearly twice as many genes with evidence of *trans*-regulatory divergence (table 1). However, we argue that the numbers of genes displaying evidence for *trans*-regulation here is an overestimate for three reasons. First, as mentioned above, the inclusion of multiple parental genotypes will induce false positives in cases where intrapopulation variation in gene expression is substantial. Second, it is likely that the difference in power between the methods to detect *cis-* and *trans* effects contributes to the number of genes detected in each category (Graze et al. 2009; Coolon et al. 2014; but see Glaser-Schmitt et al. [2018]).

Third, the estimation of parental expression differences, and therefore *trans*-regulatory effects, are more error-prone due simply to the biology of our samples. We compared larval samples across two populations that develop at different rates (egg-pupation time [h]: CI = 275.20 ± 5.48; SON = 315.21 ± 5.96; Benowitz KM, Unpublished data), making it impossible to guarantee that sampling occurred at precisely the same exact developmental stage. Thus, some ontogenetic or environmental variation in gene expression is inevitable here. The usage of multiple genotypes should mitigate this problem but is unlikely to resolve it completely, and thus we may be generating false positives for this reason. For example, we find significant and consistent population differences in expression of two fat body proteins (*Fbp1; Fbp2*) and three larval serum proteins (*Lsp1beta; Lsp1gamma; Lsp2*) that are all clearly attributed to *trans*-regulatory evolution (table 2). Fat body proteins and larval serum proteins interact in a key pathway for nutritional storage prior to pupation (Burmester et al. 1999), and therefore could lead us to a hypothesis of adaptation via *trans*-regulation to variable nutritive environments. However, it is also well established that all four of these genes undergo rapid increases in expression during the third-instar wandering stage (Burmester et al. 1999). Our results are therefore equally consistent with the possibility that the expression differences were due to slight variations in the developmental stages sampled, and that expression patterns of these genes have not meaningfully evolved at all. In contrast, the estimation of *cis*-regulatory effects is completely controlled for any such environmental variability because it is measured within individuals (Pastinen 2010), and is therefore inherently less prone to similar errors.

### Integrating Regulatory Data to Address Evolutionary Hypotheses

Recent work has sought to identify the evolutionary and structural properties associated with genes evolving via *cis*- and *trans-*regulation. Here, we demonstrate that in *D. mojavensis* larval brain *cis*-regulated genes tend to display faster rates of coding evolution. Furthermore, we show that *cis*-regulated genes also tend to occupy less central positions within transcriptional networks, confirming the results of Yang and Wittkopp (2017) and supporting their generality. Notably, we reached this conclusion using *D. melanogaster* network data, given that similar data are unavailable for *D. mojavensis*. Thus, our results suggest that gross network properties may be conserved across significant lengths of evolutionary time. Considered together, our findings linking regulatory type to evolutionary rate and network connectivity indicate that the genes experiencing *cis*-regulatory evolution are relatively unconstrained compared with *trans*-regulated genes. This perhaps contrasts with our predictions, which were that *cis*-regulation should be predominant due precisely to the presence of such constraints. However, it is not clear whether errors regarding the determination of *trans*-regulation discussed above may be obscuring any potential statistical relationships.

Ideally, the determination of regulatory evolution will also help identify adaptively regulated genes (Fraser 2011; Delbare and Clark 2018). RNA-seq experiments in ecology and evolution nearly always result in hundreds if not thousands of DE genes, many of which are likely false positives (Todd et al. 2016; Bengston et al. 2018). Truly DE genes must have experienced *cis*- or *trans*-regulatory evolution; therefore, the corroboration provided by a statistically significant regulatory effect estimated from an independent sample may help weed out noisy or environmentally variable genes. Thus, allele-specific expression data have been used to supplement studies of gene expression adaptation at the genome-wide (Juneja et al. 2016; Verta and Jones 2019) and candidate gene (Bendesky et al. 2017) levels. We thus turned our attention to the identities of the genes displaying clear patterns of both divergence and regulation, and compared between those displaying *cis*- and *trans*-regulation.

Previous transcriptomic (Matzkin et al. 2006; Matzkin 2012; Matzkin and Markow 2013; Smith et al. 2013) and genomic (Allan and Matzkin 2019) investigations of *D. mojavensis* have identified detoxification and chemosensory genes as important classes of genes likely related to adaptation to the alternative chemical environments provided by their hosts. Taking this as an a priori hypothesis, we examined the identities of genes identified here to search for candidates fitting these categories. We are most interested in the *cis*-regulated genes, which have the cleanest interpretations in this data set. Among the 33 *cis*-regulated candidate genes are 28 with *D. melanogaster* orthologs, of which 12 have described functions. Noteworthy among these is *GstD1*, a detoxification gene with considerable evidence for a functional role in adaptation across *D. mojavensis* populations (Matzkin et al. 2006; Matzkin 2008). Here, we find that expression differences in *GstD1* are clearly attributable to *cis*-regulatory evolution between these populations, leading to increased expression in the Catalina Island population. Four other genes, including three cytochrome p450s and one UDP-glycosyltransferase, have well-characterized roles in detoxification of plant chemicals as well (Heckel 2014). We also find a single chemosensory gene, *Obp99a.* Among the 19 characterized genes displaying *trans-*regulation, we find the detoxification gene *GstE1* as well as the chemosensory genes *Obp99a* (regulated in *cis* and *trans*) and *Obp99b*. Thus, although we are less confident in the *trans*-regulated gene set as a whole, this confirmation suggests that the regulatory evolution of at least a subset of these genes is accurately represented in this data set.

Although it is unsurprising that chemosensory genes are expressed and have evolved specifically in the brain, it is not as immediately clear why the expression of detoxification genes should be important in brain tissue. Detoxification is usually associated with tissues such as the midgut, Malpighian tubule, and fat body (Chung et al. 2009) and the blood–brain barrier tends to shield the brain from harmful chemicals (Stork et al. 2008; Hindle and Bainton 2014). However, the *Drosophila* blood–brain barrier is not completely impermeable to xenobiotics (Zhang et al. 2018), and important detoxification processes have been demonstrated in the brain in other insects (Zhu et al. 2010). Thus, it is at least plausible, given the chemical cocktail that *D. mojavensis* is exposed to within organ pipe and prickly pear necroses (Kircher 1982; Starmer and Phaff 1983), that some compounds may enter the brain. Alternatively, it is possible that we are witnessing the consequences of indirect selection. For example, strong selection on *GstD1* expression in the midgut might have resulted in a *cis*-regulatory change to a binding site for a transcription factor that is also highly expressed in brains. If the resulting change in brain expression is neutral or nearly neutral it may then persist without having any adaptive function.

Given the ability of our approach to recapitulate a priori hypotheses about expression evolution, we also asked whether this approach might lead to novel predictions about phenotypic and genetic adaptation. Among the remaining *cis*-regulated genes are *photorepair* (*phr*) and *CG5316* (ortholog of human *aprataxin*), which function to repair UV-damaged DNA (Boyd and Harris 1987; Hirano et al. 2007). In *D. melanogaster*, selection has generated adaptive differences in DNA repair mechanisms between tropical and temperate populations, and has resulted in both coding and noncoding genetic changes (Svetec et al. 2016). Here, both *phr* and *CG5316* are upregulated in the Sonoran Desert population, where presumably intense UV exposure is a more pressing environmental challenge than in the cooler and wetter clime of Santa Catalina Island. Interestingly, *phr* was not among the *D. melanogaster* candidate genes differentiated in sequence or expression among populations, whereas *CG5316* showed evidence of protein-coding but not expression evolution (Svetec et al. 2016). Thus, even when the predictability of DNA repair evolution pathways to similar environmental variables may extend to the gene, the type of genetic change itself may still be unpredictable.

Broadly, our usage of a replicated, tissue-specific data set and requirement of gene to display a clear regulatory pattern, especially one of *cis*-regulation, has led us to a manageable set of a few dozen highly intuitive genes that may be adaptively regulated across *D. mojavensis* populations associated with local ecological conditions. We propose that deeper understanding of patterns of regulatory evolution in ecological model systems, where there are strong predictions regarding selection, will be essential for a robust understanding of the differing roles of *cis*- and *trans*-regulation in local adaptation and evolution. 

## Supplementary Material

Supplementary data are available at *Genome Biology and Evolution* online.

## Supplementary Material

evaa145_Supplementary_Data

## Notes

**Data deposition:** This project has been deposited at the NCBI Sequence Read Archive under the Bioproject accession PRJNA574480.
