# Supplementary figures and images for: Contributions of cis- and trans-Regulatory Evolution to Transcriptomic Divergence across Populations in the Drosophila mojavensis Larval Brain
Source: Genome Biol Evol. 2020 Jul 11;12(8):1407–18. doi: 10.1093/gbe/evaa145 (PMC7495911; doi:10.1093/gbe/evaa145)

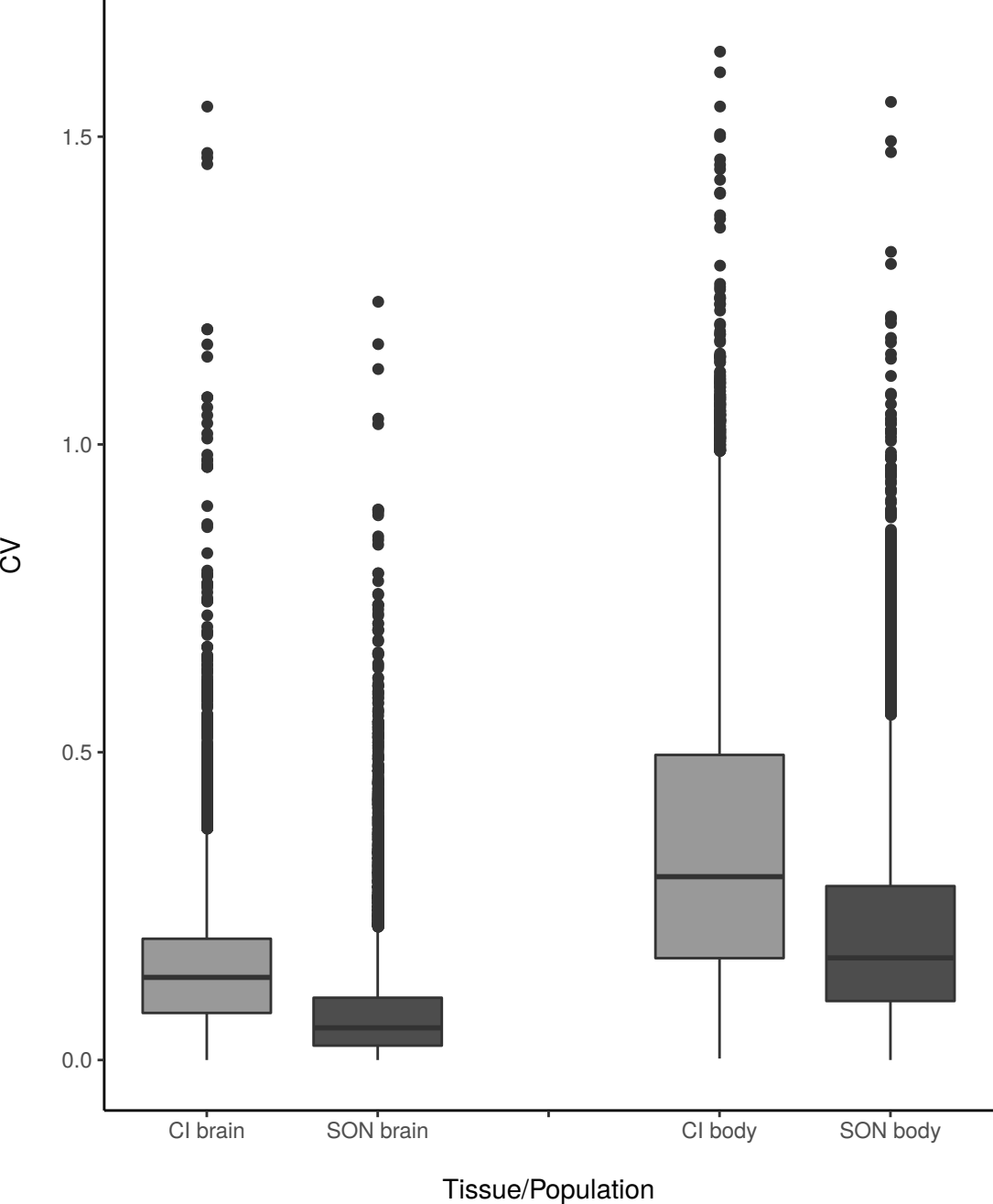

Supplement: evaa145_Supplementary_Data [file evaa145_supplementary_data.zip › gbe_supplementary_fig_S1.pdf]
